# Theoretical Studies of Homogeneous Catalysts Mimicking Nitrogenase

**DOI:** 10.3390/molecules16010442

**Published:** 2011-01-10

**Authors:** Jacopo Sgrignani, Duvan Franco, Alessandra Magistrato

**Affiliations:** 1 CNR-IOM-DEMOCRITOS National Simulation Center at SISSA, via Bonomea 265, Trieste, Italy; 2 International School for Advanced Studies (SISSA/ISAS), via Bonomea 265, Trieste, Italy

**Keywords:** nitrogen fixation, nitrogen reduction, biomimetic catalyst, Density Functional Theory, DFT, reaction mechanisms

## Abstract

The conversion of molecular nitrogen to ammonia is a key biological and chemical process and represents one of the most challenging topics in chemistry and biology. In Nature the Mo-containing nitrogenase enzymes perform nitrogen ‘fixation’ via an iron molybdenum cofactor (FeMo-co) under ambient conditions. In contrast, industrially, the Haber-Bosch process reduces molecular nitrogen and hydrogen to ammonia with a heterogeneous iron catalyst under drastic conditions of temperature and pressure. This process accounts for the production of millions of tons of nitrogen compounds used for agricultural and industrial purposes, but the high temperature and pressure required result in a large energy loss, leading to several economic and environmental issues. During the last 40 years many attempts have been made to synthesize simple homogeneous catalysts that can activate dinitrogen under the same mild conditions of the nitrogenase enzymes. Several compounds, almost all containing transition metals, have been shown to bind and activate N_2_ to various degrees. However, to date Mo(N_2_)(HIPTN)_3_N with (HIPTN)_3_N= hexaisopropyl-terphenyl-triamidoamine is the only compound performing this process catalytically. In this review we describe how Density Functional Theory calculations have been of help in elucidating the reaction mechanisms of the inorganic compounds that activate or fix N_2_. These studies provided important insights that rationalize and complement the experimental findings about the reaction mechanisms of known catalysts, predicting the reactivity of new potential catalysts and helping in tailoring new efficient catalytic compounds.

## 1. Introduction 

A long-standing holy grail of chemistry is the fixation of molecular nitrogen. Nitrogen is ready available in the air, but due to its triple bond, its non polarity and to its large HOMO-LUMO gap this molecule is highly inert and its use as a feedstock is challenging [1,2,3].

**Figure 1:**
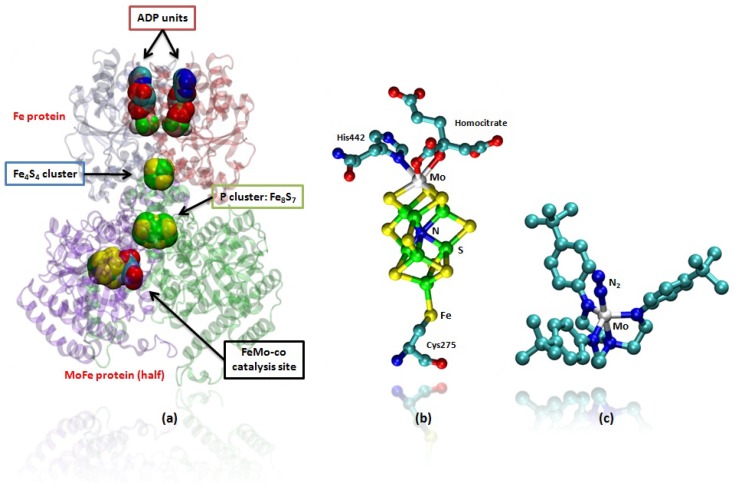
**(a)** Mo containing nitrogenase enzyme (PDB code: 1N2C), only half of the protein is shown for clarity. In the upper part the two chains of the Fe protein are shown in which the adenosine diphosphate (ADP) units and the Fe_4_S_4_ cluster are represented in van der Waals spheres. These are involved in the electron transfer to the catalytic unit. In the lower part of the picture is represented the MoFe protein where are placed the P-cluster and the FeMo-co, which mediates electron transfer and which performs N_2_ fixation, respectively. In (**b**) FeMo-co of the nitrogenase is shown; In (**c**) a ball and stick representation of the Schrock’s Mo catalyst is provided. Hydrogens are omitted for clarity.

In Nature the highly specialized enzymes nitrogenases, expressed by soil bacteria, perform the important function of fixing N_2_, converting the nitrogen present in the atmosphere into ammonia under mild conditions of temperature and pressure [4]. In the Mo containing nitrogenase this function is performed at an iron-molybdenum cofactor cluster (MoFe_7_S_9_) usually referred to as FeMo-co (Figures 1a and b), where the electrons for the activation of N_2_ are transferred by the Fe protein, which contains a Fe_4_S_4_ cluster, and by a P cluster, which contains a Fe_8_S_7_ unit (Figure 1a) [4,5]. 

Despite the large number of experimental and theoretical studies the detailed mechanism by which the enzyme fixes N_2_ under ambient conditions is not clear [4,5,6,7,8,9,10,11,12,13,14,15,16]. The synthesis of ammonia is, however, important for the industrial production of fertilizers, crucial for sustaining the World’s agricultural production. Ammonia is obtained, industrially, via the Haber-Bosh process on the surface of a heterogeneous iron catalyst under drastic temperature and pressure conditions of 500 °C and 150-200 atm. This process accounts for the production of over 100 millions of tons of ammonia annually. Nevertheless, it uses about 1% of human energy consumption, raising several economic and environmental issues [2,4]. Therefore, inorganic chemists have been occupied for decades to find efficient biomimetic catalysts able to perform the same function of nitrogenases under the same mild conditions [3]. Unfortunately, cubane clusters that chemically resemble the enzymes exhibit only a modest reactivity towards N_2_ activation [1]. Instead, mono or dinuclear transition metal complexes can activate N_2_ to various degrees [17]. After the discovery of the first transition metal-N_2_ complex, [Ru(N_2_)(NH_3_)_5_]^2+^, a large number of metal complexes coordinating N_2_ has been isolated for almost all transition metals [18]. These include, among many others, the first Mo-N_2_ complex, *trans*-[Mo(N_2_)_2_(Ph_2_PCH_2_CH_2_PPh_2_)_2_], or the mono and dinuclear ruthenium-sulfur (Sellmann-type) complexes [17,18,19,20]. 

**Figure 2:**
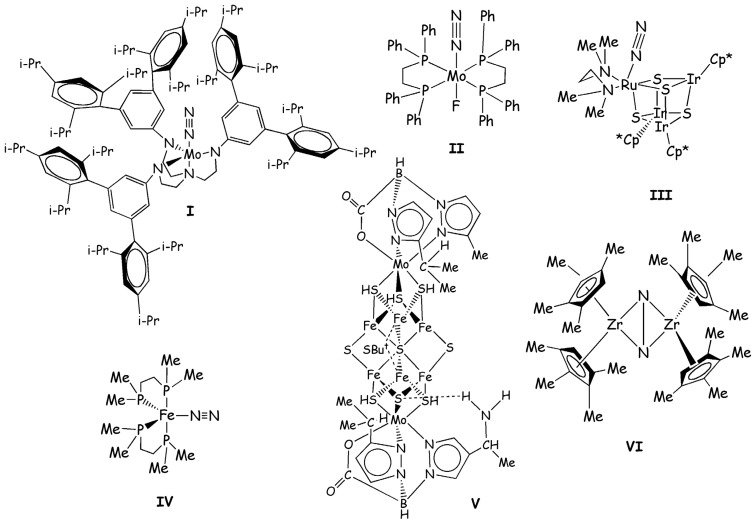
Inorganic compounds discussed in this review. i-Pr, Me, Ph, and Bu^t^, Cp* abbreviations stand for isopropyl, methyl, phenyl, tert-butyl, and pentamethylciclopentadienyl substituents, respectively.

However, only in 2003 the Mo(N_2_)(HIPTN)_3_N (with (HIPTN)_3_N=hexaisopropyl-terphenyl-triamidoamine) catalyst (**I**, Figure 2) has been proven to convert nitrogen into ammonia with an overall yield of 65% [21]. This is so far the only inorganic compound able to perform this function catalytically under ambient conditions and in the presence of a suitable proton and an electron source. Hence, it represents a breakthrough in the efforts to find a homogeneous catalyst for N_2_ fixation. Due to the importance of **I**, a large number of experimental and theoretical studies has been carried out to characterize in detail its catalytic mechanism [3].

The number of inorganic catalysts synthesized with the aim of activating molecular nitrogen is impressive [17] and among these only some complexes can produce NH_3_ from N_2_. In this review we focus: (i) on theoretical studies performed to understand the reaction mechanisms of the compounds for which NH_3_ production has been observed experimentally; (ii) On calculations performed to predict if compounds which activate N_2_, would perform nitrogen fixation with the same mechanism as **I** (Schrock’s mechanism) or via direct N_2_ hydrogenation; (iii) On computational studies performed to assess the performance of the catalysts depending on the ligands and transition metals employed (computational design of new catalysts). Therefore, the aim of this review is to show how the theoretical studies can be a crucial help to understand and predict reaction mechanisms, providing a detailed picture at atomistic level of the intermediates involved in the catalytic cycles and unveiling the electronic and structural properties of real or potential nitrogen fixing catalysts.

## 2. Density Functional Theory in the Study of Inorganic Catalysts

Facing the problem of nitrogen activation is a challenge from the experimental point of view. In fact, understanding the reaction mechanism of the biological systems or their biomimetics, as well as the electronic, structural and thermodynamic properties that drive the enzymatic/catalytic reactions can be difficult or not accessible to experiments. Theoretical methods, and in particular Density Functional Theory (DFT), have been of valuable help to predict the reactivity of both transition metal enzymes and catalysts, assessing which is the most likely reaction path (when multiple paths are possible), determining the structures of reactants, of (known or unknown) intermediates, products, transition states and elucidating the structural electronic properties of all these species [22,23,24]. 

A rigorous description of DFT is already available in many reviews [25,26,27,28,29]. Hence, here we only provide the basic concepts of the method and we highlight the problems faced by DFT calculations when dealing with inorganic catalysts. DFT describes the electronic structure of atoms and molecules in terms of the electron density (ρ) of the N-electron system. In contrast to wavefunction methods (such as Hartree-Fock (HF) and post-HF), ρ is the central quantity in DFT as the ground state energy of the system is a unique functional of ρ. An exact expression of the total energy as function of ρ is unknown. Kohn and Sham (KS) solved this problem by replacing the true kinetic energy of the real N-electron system with that of a system of non-interacting N-electrons. Within the KS formulation the central issue becomes the expression of the exchange-correlation (XC) functional term as a function of ρ. In fact, no exact analytical form of this exists [27]. Different approximations have been proposed to account for this problem and the quality of the DFT results is strictly related to the functional used to evaluate the XC energy contribution. Among the different approximations, one of the most popular is gradient-corrected one, in which the XC term depends on ρ as well as on its gradient. Typical examples of this are the BLYP [30,31] BP [30,32], PBE [32,33] and PW91 [34] functionals. These account for both the electron correlation and the exchange contributions in an approximate manner. However, an improvement to the accuracy of the gradient-corrected XC functionals has been achieved by combining the DFT-exchange functional term with the Hartree-Fock exact exchange. This approach led to the development of hybrid functionals, among which B3LYP is the most popular [31,35,36]. Many other types of functionals have been created over the years by different combinations of the DFT exchange term with the exact HF exchange [27]. However, none of these hybrid functionals solves all the issues associated to the treatment of transition metal compounds. Furthermore, their use is still not accessible to codes, which perform periodic calculations since they are too computationally expensive to be used with plane waves basis sets. The choice of the most appropriate XC functional for the system in study is, nevertheless, fundamental as this usually affects the energetics (thermodynamic stabilities and free energy barriers) of the catalytic reactions [37], redox potentials [38], calculated spectroscopic properties etc. to a very large extent. In contrast, the structural properties of these molecules are usually not markedly affected by this choice.

Another problem, common to all XC functionals, is the self interaction [39]. This effect arises from the approximate form of the exchange term that does not totally cancel the interaction of an electron with itself. Consequences of this are, among others, that the eigenvalue of the highest occupied Kohn-Sham orbital does not correspond to the ionization potential and unphysical delocalization of the electron density may occur in open shell systems, as frequently transition metal compounds are [39]. Although different approaches have been proposed to correct this error, they are usually quite demanding from the computational point of view [27]. 

An issue that is strictly connected with the theoretical treatment of inorganic complexes is an accurate description of correlation [27]. In fact, transition metals present partially filled valence d shells, which result in a number of nearly degenerate electronic states (static correlation). In addition, correlation effects arise also from electron-electron repulsion at short range (dynamical correlation). Correlation is completed neglected in Hartree-Fock formalism and it would be necessary to consider multireference systems to account for it [27]. However, accurate correlated methods are highly computationally expensive and their use is hampered already in the treatment of medium size inorganic molecules. By contrast, DFT, although being a single reference method, accounts for correlation effects in an accurate and computationally efficient manner, but the accuracy of the calculations markedly depends on the XC functional used. 

Finally, DFT does not account for dispersion forces, which are at the basis of π-stacking or hydrophobic interactions. These may be important to reproduce the structural and energetic properties of inorganic catalysts [40,41,42]. This issue has been only recently addressed by some hybrid functionals [42,43,44].

DFT is computationally quite efficient and it is used for systems of up to thousands of atoms [45]. A transition metal catalyst is usually sensibly smaller, but if one wants to individuate the most likely catalytic path, several possible intermediates and transition states have to be considered. This search can be computationally demanding even for a medium size catalyst. Hence, a reduced model is most commonly used, after verifying that it reproduces the main electronic and structural features of the real catalyst. Sometimes a hybrid quantum-classical (QM/MM) approach is also introduced [46]. In this approach part of the catalyst is treated at QM level, while the remaining part is considered at force field MM level [23,47]. Furthermore, if one wants to consider explicitly the solvent in which the catalytic reaction occurs the number of atoms increases rapidly. The most common approach adopted in the quantum-chemistry community to address this aspect is the use of implicit solvation models, in which the solvent is represented as a continuum medium [48,49,50,51,52]. These models allow estimating the solvation free energies in a computationally efficient manner. Yet, this approach is not appropriate if the solvent is directly involved in the reaction or if hydrophobic or hydrogen bonds interactions contribute to the reaction energetics [42]. An alternative to this is the use of QM/MM methods in which the catalyst is described quantum mechanically, while the solvent is treated at the MM level [28,53].

In practice, to perform DFT calculations a careful comparison with experimental data (and/or higher-level calculations, when experimental data are not available) is essential to establish the reliability and predictability of the adopted computational protocol. Once this aspect is verified, DFT calculations can complement experimental findings and predict structural, kinetic and thermodynamic properties of the systems studied [22]. Nowadays DFT represents the best compromise between accuracy and efficiency, and it is hence the theory of choice to study the electronic properties and the reactivity of transition metal catalysts. 

## 3. N_2_ fixation by Mo Catalysts 

To date, molybdenum complexes are the only organometallic compounds able to catalyze the reduction of molecular nitrogen to ammonia under mild conditions. In particular, MoN_2_((HIPTN)_3_N) (**I**, in Figure 2), discovered by Yandulov and Schrock in the 2003 [21], can produce ammonia with a catalytic yield of 60-65% under ambient conditions, in heptane media, using 2,6-dimethylpyridinium (lutidinium, LutH^+^) and decamethylchromocene [(Cp^*^)_2_Cr] as proton and electron sources, respectively. For its good and exclusive catalytic performances among all other inorganic catalysts synthesized to date, **I** represents a milestone in the research of new methods for the synthesis of ammonia. The only other catalytic Mo compound known to date produce in fact hydrazine and not ammonia as final product [54].

In recent years a large number of experimental [55] and theoretical [56] investigations have been carried out to rationalize the catalytic mechanism of **I** [42,57,58,59,60,61]. However, some important details of this reaction are still controversial. In this section we will provide an overview of the theoretical investigations performed so far on this topic.

**Scheme 1:**
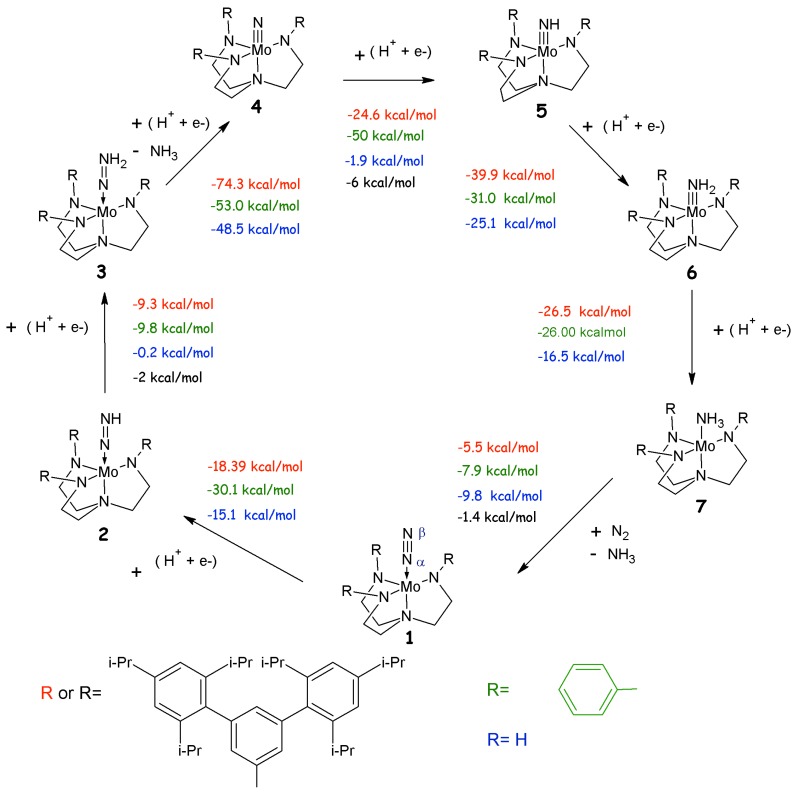
Reaction mechanism of the Schrock’s catalyst. Reaction energies (kcal/mol) are reported as a sum of consecutive protonation and reduction steps. In the figure the protonated intermediates, referred in the text with the same number of the corresponding neutral compound followed by a +, are not shown. The colours correspond to theoretical studies in which different approximations of R have been used; namely R=H [62], R=Ph [42] and R=HIPT [58]. Available experimental free energies are reported in black.

Since several reaction intermediates have been isolated by Schrock *et al.* [21], a mechanism has been proposed (Schrock’s cycle, Scheme 1) in which the reaction proceeds via the stepwise addition of protons and electrons, that, in six catalytic steps, leads to the release of two ammonia molecules. According to the Schrock’s cycle the reaction proceeds from the MoN_2_ (**1**,Scheme 1) via the addition of a proton to the Nβ of the dinitrogen unit. This is followed by a one-electron reduction, leading to the formation of a diazenido complex **2**. The reaction proceeds with a protonation and subsequent reduction with formation of a positively charged and neutral hydrazido intermediates, respectively (**3**, Scheme 1). An additional protonation leads to a positively charged hydrazinium intermediate **4^+^**. From this compound the first ammonia is released and the subsequent reduction results in the formation of a nitrido complex **4**. The following stepwise additions of protons and electrons lead to the formation of imido, amido and amine compounds **5-7**. The calculations reveal that the energetically most difficult steps are the conversion of MoNNH to MoNNH_2_ and of MoN to MoNH [3]. Finally, the regeneration of **I** goes through a direct coordination of N_2_ to the MoNH_3_, avoiding the formation of a naked intermediate [3]. In spite of a large amount of experimental data available for this catalytic cycle, the theoretical calculations on this topic still remain challenging. 

A complete discussion of the theoretical results found for every step of the catalytic cycle has been recently and accurately reviewed by Schrock [3] and Shenk *et al.* [58]. In these papers every reaction step is discussed in view of the theoretical and experimental data available in the literature. Therefore, we will not discuss this aspect in detail here, but we would rather highlight how different approaches, used within the DFT framework, have addressed different aspects of this key catalytic process. Hopefully, this will give the reader a flavour of the utility and the difficulties emerged in applying DFT methods to the study of the Schrock’s catalyst.

A general agreement is found concerning the geometries of the different intermediates for all the different computational protocols employed so far. The geometrical properties of the compounds are in good agreement with the experimentally determined ones also when different XC functionals are used. Nevertheless, the most debated aspect of the DFT investigations on the Schrock’s cycle concerns the calculation of reaction (free) energies of the various steps. Remarkably, appreciable discrepancies have been found for the ΔH/ΔG estimated with different computational protocols (see Scheme 1). In fact, every group addressed this problem using a different computational approach. Studt *et al.* [62] calculated the reaction free energy differences for each step of the catalytic cycle using B3LYP [31,35,36] and considering a reduced model of **I**, where the HIPT groups are replaced by H atoms. However, this model is too simplified to correctly account for the reaction energies. In fact, Le Guennic *et al*. [63] observed that the geometry of the triamidoamine ligand is not reproduced correctly in the amine intermediate. This prompted the use of other models of the catalyst where the HIPT substituents were simplified less drastically. In particular, Le Guennic *et al.* [63] tested models of increasing complexity replacing HIPT with a CH_3_, a 3,5-(C_6_H_2_)_2_C_6_H_3_ and a 3,5-(2,4,6-Me_3_C_6_H_2_)C_6_H_3_. In all these compounds the geometry of the amine intermediate was correctly reproduced and, thus, these models appear to be more reliable with respect to the most simplified one. Their computational investigation, carried out employing B3LYP [31,35] and BP [30,32], shows significant differences in the energy between the most simplified and the larger models, in particular for the step involving NH_3_/N_2_ exchange [63]. 

In a different paper Cao *et al.* [60] replaced the HIPT with a phenyl and they carried out a characterization of the reaction mechanisms on both the Schrock’s catalyst and the nitrogenase enzyme. This study, carried out with BLYP [30,31] and PW91 [33], assessed that the energetics of the biological and the inorganic processes were similar. However, the proton and electron sources considered in this study are different from those used experimentally by Yandulov *et al.* [21] and this prevents a comparison with other theoretical data. 

Subsequently Magistrato *et al.* [42] investigated the catalytic reaction using static and dynamic (Car-Parrinello, CP) [64] within a BP-DFT [30,32] approach. In these calculations each HIPT is replaced by a phenyl, after testing via QM/MM calculations that the reduced model correctly reproduces the structural features of **I**. This type of model, previously proposed also by Cao *et al.* [60], represents a good compromise between accuracy and computational efficiency.

As in previous studies, Magistrato *et al.* observed remarkable discrepancies between calculated and experimental reaction free energies [42]. Therefore, for those steps for which ΔG was known experimentally they also performed calculations with different computational protocols, namely different localized basis sets (6-31+G-(d) or 6-311++G(d,p)) and different XC functionals (B3LYP, [30,31,35] B3P [30,32,35], PW91 [33], BHandHLYP [30,35], and BHandH [30,35]). This allowed testing the influence of the level of theory on calculated reaction (free) energies of the Schrock’s cycle.

Moreover, Magistrato *et al.* accounted also for the effect of explicit solvent on the reaction free energies. In addition to a continuum solvation model, used also in previous studies, they adopted a micro solvation approach, including discrete number of solvent molecules (up to four). The inclusion of explicit solvent reduces the errors of the reaction free energies for protonation steps within the limit of DFT accuracy. This improvement is due to hydrophobic interactions present between the catalyst and the solvent that are totally neglected in the implicit solvation model. In contrast, the micro solvation approach has no effect on the discrepancies observed for the ΔG of the reduction steps [42]. These latter, in fact, seem to be particularly sensitive to the choice of the XC functional and, in this respect, B3LYP [31,35,36] represents the best choice. Most importantly, the comparison of the different computational protocols employed in this paper gives an important picture of the accuracy and reliability of DFT in this kind of theoretical investigations.

Alternative reaction pathways have been also proposed with respect to that originally formulated by Yandulov *et al*. [21]. In particular, Reiher *et al.* have accurately investigated the first proton addiction, as this appears to be an energetically crucial step for the catalytic cycle. In principle, the formation of the neutral diazenido complex can occur via a protonation and subsequent reduction of Mo-N_2_ or vice versa. DFT calculations, in which the two possibilities have been considered, show that the protonation is more feasible before the reduction simply for thermodynamic reasons and this feature is common to all subsequent steps of the catalytic cycle [58].

Moreover, Schenk *et al*. [58], employing as a model the complete catalyst, considered a possible alternative route in which the protonation does not occur directly on the substrate, but rather the proton is transferred to one of the equatorial N atoms of the (HIPTN)_3_N ligand and only after reduction it migrates on Nβ of the substrate. This mechanism is the preferred one for the formation of MoNNH (**2**) and MoNNH_3_^+^ (**4^+^**), while for MoNNH_2_ (**3**) the direct protonation of the Nβ group is preferred by more than 12 kcal/mol. In addition, for **3** different MoN_2_H_2_ isomers have been considered [58]. DFT calculations show that the *cis* and *trans*-hydrazido isomers are only 9.1 kcal/mol and 4.2 kcal/mol higher in energy than the isodiazene structure, respectively. This does not allow excluding that a side reaction, involving the formation of these isomers, may take place. 

Interestingly, theoretical evidences highlight that, when the H atom arrives in the vicinity of the N_2_H_2_ group, the first NH_3_ starts leaving the catalyst. This reaction can take place even in absence of a reductant. In the fourth, the fifth and the sixth steps the protonation occurs directly on the Nα atom of the substrate [58]. At variance with previous works, these authors have adopted also a ‘one-pot model’ for the protonation steps in which acid/base molecules have been considered simultaneously in the calculations. This approach did not show any significant energetic difference from the isolated molecule one. The very careful and detailed computational study performed by Schenck *et al.* [58] reproduces experimental reaction free energies quite accurately. Therefore, the authors attributed the discrepancies observed in previous theoretical works mainly to the use of reduced model systems.

A crucial step for the development of an efficient Mo catalyst is the substitution of NH_3_ in the amine complex with a new N_2_ to regenerate the catalyst and start a new catalytic cycle. In fact, a too strong or too weak binding of the two molecules can drastically influence the turnover of the catalyst as well as its quality (catalyst poisoning) [57].

From the experimental point of view the ΔG for the NH_3_/N_2_ exchange requires 1.4 kcal/mol. This step has been investigated by different groups, but the experimental reaction free energy has been always overestimated by more than 4 kcal/mol [42,57,58,62]. 

Recently, this step has been also investigated by Schenk *et al*. [57] using CPMD. In this work the authors studied the NH_3_/N_2_ exchange in the neutral form of the catalytic complex. They have supposed two trajectories for the approach of N_2_ and for the release of NH_3_. The two trajectories consist of a route on the plane determined by the three equatorial amide atoms of the triamidoamine ligand, and of a second route from the axial position occupied by the substrate. Following the two trajectories, the authors identified a six-coordinated intermediate with N_2_ and NH_3_ in the equatorial and axial position, respectively. A calculation of the infrared spectra (IR) has been done to test whether the Mo(NH_3_)(N_2_) intermediate could be experimentally detected. The comparison of experimental and calculated IR spectra of the MoN_2_ complex assesses the good accuracy of the calculations, suggesting that, in principle, the formation of the six-coordinated intermediate could be monitored experimentally observing the N-N stretching mode near 2,000 cm^-1^. Finally, Schenk *et al*. have performed a computational design of peripheral ligands to increase the efficiency of the Schrock-type catalyst, identifying new potential candidates, which gave promising computational results [59].

**Scheme 2:**
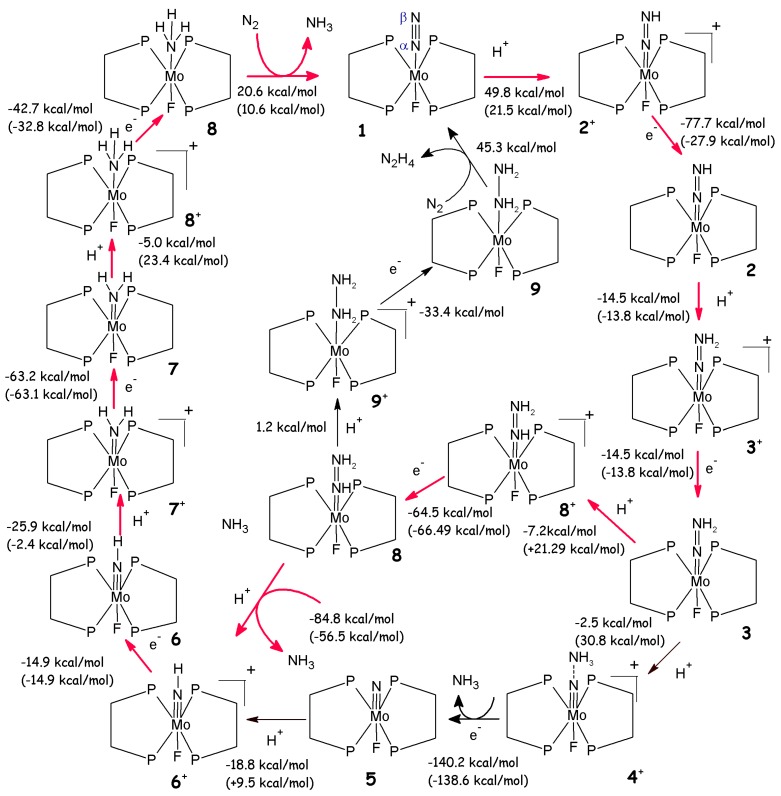
Chatt’s reaction mechanism. The most likely reaction path is highlighted with red arrows. Reaction free energies calculated considering HBF_4_ in Et_2_O as proton source are reported, while, in parenthesis, free energies calculated considering lutidinium as proton source are also given.

The detailed characterization of the Schrock’s catalytic cycle has also revitalized the interest towards similar reaction mechanisms. Historically Schrock’s cycle was anticipated by Chatt’s cycle (Scheme 2), which is based on compounds of formula M(N_2_)_2_L_2_ with M=Mo or W and L=1,2-bis(diphenylphosphino)-ethane=dppe (**II** in Figure 2) or 1,2-bis(diethylphosphino)-ethane=depe. These compounds did not undergo a true catalytic cycle.

Interestingly, Stephan *et al.* have investigated the energetic profile of Chatt’s cycle considering a Mo complex in which the real phosphine ligands are replaced by H_2_PCH_2_CH_2_PH_2_ (diphosphinoethane, dpe) (**1** in Scheme 2) and the position *trans* to N_2_ is occupied by a fluorine [65]. The authors have studied the complete reaction path for NH_3_ production and they have compared it with Schrock’s cycle [65]. Their results indicate strong similarities between the two cycles: i.e. a progressive reduction of the N-N bond order and increase of the Mo-N one, the same intermediates found in the Schrock cycle are also found and the reaction in both cycles proceeds via stepwise addition of protons and electrons, in which the first protonation is the most endergonic and the N-N bond breakage step is the most exothermic. However, in contrast to Schrock’s cycle, the reaction cannot proceed with lutidinium as proton source, since some reaction steps are not thermally accessible (Scheme 2). Therefore, a stronger protonation agent, such as HBF_4_ in Et_2_O, has to be used to allow the full conversion of N_2_ to NH_3_. In addition, the most likely reaction path (Scheme 2) differs from Schrock’s cycle since the neutral isodiazene (**3**) can be further protonated in Nα leading to **8^+^**. Subsequent electron transfer leads to MoNHNH_2_ (**8**), which upon protonation on Nβ results in an imido complex and ammonia. The rest of the reaction proceeds as in Schrock’s cycle [65]. 

A possible alternative pathway involves the formation of bis dinitrogen complexes in which F is replaced by an additional N_2_. However, binding of the second N_2_ to **1** is not thermodynamically favoured. Moreover, MoN_2_(dpe)_2_F is unstable towards disproportionation, leading to a loss of catalyst during the cycle [65]. Thus, to have a true catalytic system, this disproportionation must be avoided by, for example, introducing appropriate ligands [65].

## 4. N_2_ Fixation by Other Inorganic Catalysts

A large number of other transition metal complexes have been demonstrated to reduce molecular nitrogen to ammonia or to bind and activate N_2_ [17]. In this paragraph we concentrate on the theoretical studies of the reaction mechanisms carried out on these compounds to characterize the complete conversion of N_2_ to NH_3_.

**Scheme 3:**
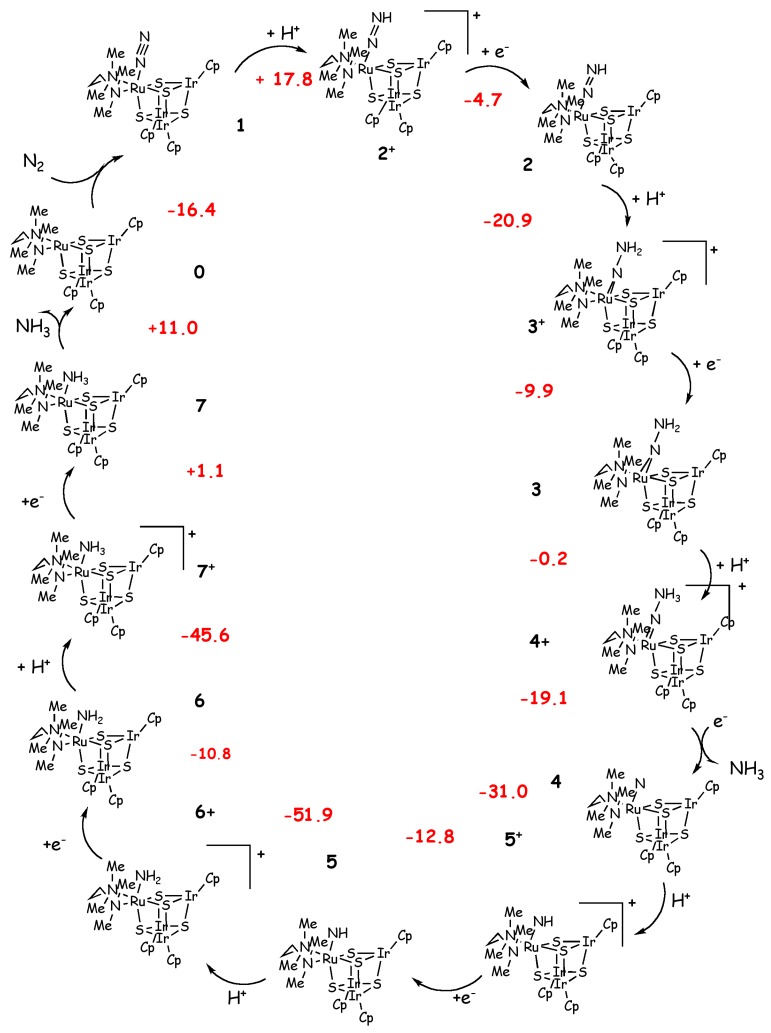
Schrock’s cycle for the Ru cluster of Mori *et al.* [18,66]. Calculated reaction energies (kcal/mol) are shown in red.

Recently Mori *et al.* synthesized a cubane-type metal sulfido cluster bearing dinitrogen as a ligand ([(RIr)_3_ Ru(tmeda)(N_2_)(*μ*_3_-S)_4_], with tmeda=Me_2_NCH_2_CH_2_NMe_2_ and R= Cp*=η^5^-C_5_Me_5_, **III**, in Figure 2) [66]. In this complex N_2_ is coordinated in an end-on manner and it is moderately activated as demonstrated by the vibrational frequency of N-N triple bond and the elongated N-N distance. Based on this important finding Tanaka *et al.* carried out a DFT-B3LYP [31,35,36] investigation of the reduction of N_2_ to NH_3_ performed by this type of cluster [18]. In this work the authors used a reduced model in which the Cp* has been replaced by a cyclopentadienyl substituent and they studied the mechanism according to the Schrock cycle (Scheme 3). 

In analogy with what is observed experimentally for **I**, the calculations show that in the first half of the catalytic cycle there is a weakening of the N-N bond, which leads to the release of the first ammonia after formation of the hydrazinium complex, and in the second part of the catalytic cycle the Ru-N bond is progressively weakened [18]. The reaction enthalpies are calculated considering lutidinium and Co(Cp*)_2_, as proton and electron sources, respectively. With the only exception of the reduction step of the amine complex, which is thermoneutral, the only two endothermic steps are the first protonation, which leads to the formation of a positively charged diazenido complex (**2+** in Scheme 3), and the dissociation of the second ammonia molecule with regeneration of the naked catalyst. This step has been demonstrated to be unlikely in Schrock’s catalyst [3,57]. Thus, the overall catalytic process is exothermic by -193 kcal/mol, which indicates that the catalytic production of NH_3_ is feasible, if compared with the calculations performed on the Schrock catalyst (-198 [62], -171, [42] -117 [58] kcal/mol). The energetic profile of this cycle shares some features with that calculated by Studt *et al.* [62] for the Schrock system. This is observed in the N_2_ binding energy (-16.4 vs -14.2 kcal/mol), the enthalpy change for the first protonation (17.8 *vs*. 21.5 kcal/mol) and the enthalpy change for the NH_3_/N_2_ exchange (-5.4 *vs.* -5.4 kcal/mol). However, as pointed out by Reiher *et al.* [56] the model of Studt *et al.* [62] is oversimplified to reproduce correctly the energetics of the catalytic cycle. Not surprisingly, the reaction energies calculated by Tanaka *et al.* are close to those of Studt *et al.* [62] as the oversimplified model used in this latter study does not account for any steric influence of the ligand on the reaction mechanism and resembles more closely the situation of N_2_ in the Ru cluster. Yet, in this latter, the NNHx ligand has been observed to interact with the vicinal Ir atom. According to the calculations performed by Tanaka *et al.* [18] **III** may be a suitable catalyst for the N_2_ reduction if a proper choice of proton/electron sources is done. 

Recently, Tanaka and co-workers presented also a computational study for different cubane-type metalsulfido clusters in which the Ru atom of **III** has been substituted with different metals (M=V, Cr, Mn, Fe, Co, Ni, Cu, Mo, Ru and W; R= Cp) [67] to propose new clusters with more efficient N_2_ activation than the original one [18]. DFT calculations suggest that MoIr_3_S_4_ and WIr_3_S_4_ may work as potential catalysts for N_2_ functionalization. 

Another interesting study concerning the reaction mechanism of compounds able to reduce N_2_ to NH_3_ was performed by Yelle *et al.* [68]. In this case, a DFT-B3LYP [31,35,36] approach was employed for studying the N_2_ fixation mechanism performed by Fe(dmpe)_2_N_2_ (**IV**, Figure 2) (with dmpe=1,2-bis(dimethylphosphino)ethane). This compound was demonstrated experimentally to produce ammonia under acidic conditions [69].

**Scheme 4:**
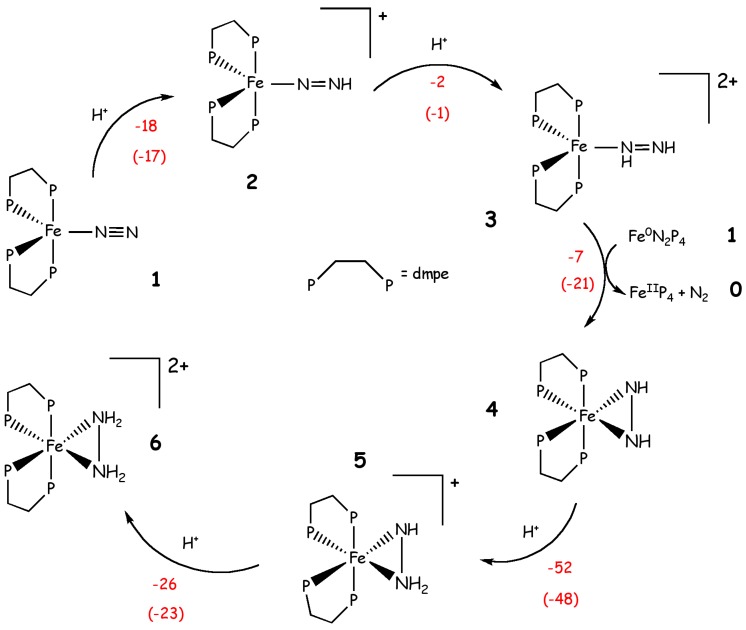
Symmetric protonation of Fe(dmpe)_2_N_2_ as proposed by Yelle *et al*. [68] Phosphine ligands are dmetylphosphinoethane (dmpe). Reaction energies (kcal/mol) and free energies, in parenthesis, are shown in red.

At variance with previous studies Yelle *et al.* identified the most probable route for the N_2_ reduction considering three possibilities: (i) an asymmetric monomeric mechanism (Chatt-like) that consists in the successive protonations at the terminal nitrogen, (ii) a symmetric monomeric mechanism, which involves the formation of diazene and hydrazine intermediates and (iii) a dimeric mechanism where the reaction occurs via the formation of a bridged N_2_ species. According to these calculations the Chatt-like mechanism is the less likely as it possesses unfavorable reduction or protonation steps. The same holds true for the dimeric mechanism, for which the formation of the initial (Fe(dmpe)_2_)_2_N_2_ dimer is endothermic, although the rest of the mechanism is characterized by several exothermic steps. Instead, the most likely pathway appears to be the symmetric protonation mechanism depicted in Scheme 6. In this mechanism, both nitrogen atoms are protonated alternatively in several steps, proceeding up to formation of an iron diazene complex (**3** in Scheme 4). 

This is subsequently reduced by **1** via a 2-electron reduction, leading to an η^2^-hydrazido intermediate **4**. Two subsequent protonations lead to η^2^-hydrazine complex **6**. The overall process is highly exothermic (ΔH = -105 kcal/mol and ΔG = -110 kcal/mol). Further protonation of **6** and release of N_2_H_5_^+^ is also exothermic (ΔH = +6 kcal/mol and ΔG = -8 kcal/mol), but the protonation of **6** with the release of ammonia is unfavorable. Experiments on similar iron complexes suggest that a disproportionation reaction may occur from **6**, leading to a mixture of N_2_H_5_^+^ and a smaller amount of NH_4_^+^. However, the exact mechanism of ammonia formation from this pathway is unclear [68]. This mechanism is energetically more favorable, but it relies on the amount of **1**, which may be in small concentrations in presence of a strong acid. Therefore, it is not possible to formulate conclusive hypotheses about the most likely reaction path.

An interesting contribution to design a potential biomimetic catalyst was done by Dance [1]. In his work Dance evaluated the catalytic activity of synthetic compounds formed by metal sulfide cubane clusters that resemble the NFe_7_MoS_9_ core of nitrogenase. On the basis of his theoretical studies carried out on the biological cluster, he established the basic ingredients that a nitrogenase mimic should have and he looked in the literature for compounds with these characteristics [1]. After selecting several systems Dance verified their potentialities as nitrogenase mimics, considering if they were able to reproduce the intermediates of the enzymatic cycle as predicted by DFT calculations [7,8,9,70]. The ligands design of these clusters is performed according to ligands available in the literature easy to obtain synthetically. Based on these calculations Dance predicts **V** (Figure 2) to be the most promising compound to perform N_2_ fixation, assuming that a suitable source of protons and electrons is given. Therefore, this interesting work can provide a guide to the experimentalists for the synthesis of clusters mimicking nitrogenase [1]. 

While the catalytic protonation of N_2_ has been achieved by Schrock, the direct N_2_ hydrogenation under mild conditions is still one of the unresolved challenges in chemistry. However, this process does not mimic the biological nitrogen fixation, which occurs via proton and electron transfer to the FeMo-co, therefore this part will be only briefly discussed here. 

Pool *et al.* showed in 2004 that η^5^-(Cp_5_Me_4_H)_2_Zr_2_(μ^2^,η^2^,η^2^-N_2_) complex (Chirik-type) with a side-on coordinated N_2_ (**VI**, Figure 2) produces ammonia in small yields, along with several Zr side products, after the addiction of five H_2_ molecules [71]. The reaction with the first two H_2_ occurs at room temperature, while the addition of the other three H_2_ molecules occurs after gentle heating. 

**Scheme 5:**
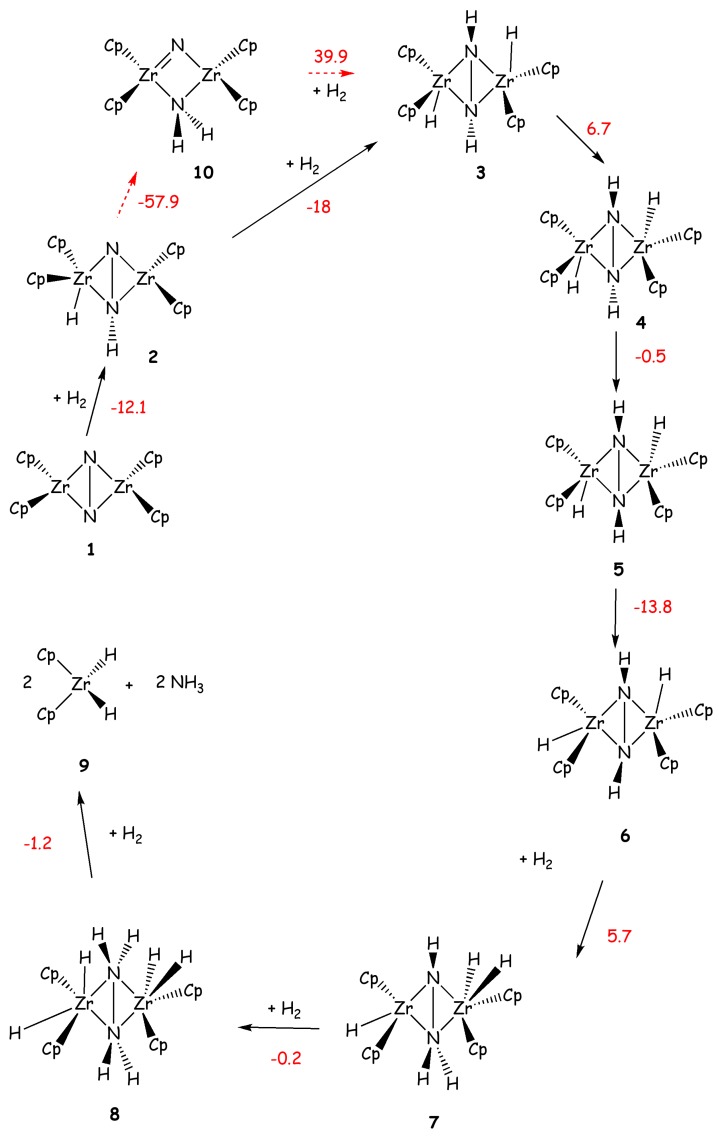
N_2_ hydrogenation performed by a model of Chirik complex. Reaction energies (kcal/mol) are shown in red. Reaction energies, calculated with respect to **1**, for the formation of **6**, **9** and **10**, using as a model the real Chirik complex, are -35.3, -44.2 and -56.7 kcal/mol, respectively.

A large amount of literature data is also available for the study of dinuclear Zr complexes, as several studies have been carried out on this complex and on similar precursors [72,73,74,75,76]. For **VI** Miyachi *et al.* have carried out a B3LYP [31,35,36] -DFT study showing that the calculated barriers for the first four additions of H_2_ are 20.4, 10.9, 24.1 and 28.0 kcal/mol, respectively [72]. The reactions involve multi centers transition states including the added H_2_, the two Zr and the two N atoms. The final addition of H_2,_ leads to the dissociation of the catalyst in two subunits and to the release of two NH_3_ molecules (Scheme 5). A comparison of the reaction mechanism of this compound with its precursors of formula P_2_Ν_2_Zr(μ^2^,η^2^,η^2^-N_2_)Zr(P_2_N_2_) with P_2_N_2_=PhP(CH_2_SiMe_2_NSiMe_2_CH_2_)_2_PPh and Ph=phenyl (Fryzuk-type) was also done.

The latter complex was shown experimentally to react with only one H_2_ molecule. Theoretical studies have proved that the difference between the two catalysts depends on the rigidity of the ligands, which results in different intermediates after the first H_2_ addition. The intermediate obtained for the Fryzuk-type complex requires larger activation energy for the second H_2_ addiction with respect to the Chirik-type complex (ΔH^#^ = 11 *vs.* 24 kcal/mol or ΔG^#^ = 16 *vs.* 24 kcal/mol), rationalizing the different experimental findings [72,74].

**Scheme 6:**
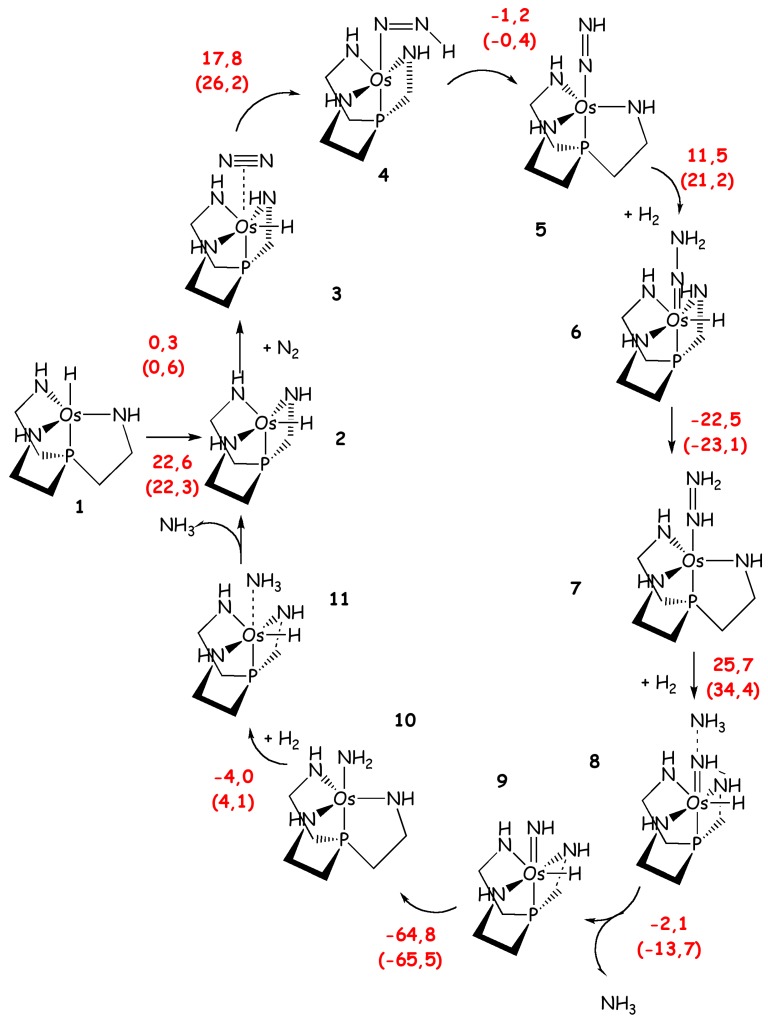
Catalytic cycle of Os modified Schrock’s catalyst as proposed by Holsher *et al.* [77]. Reaction energies, and free energies in parenthesis (kcal/mol), are shown in red.

Hoelshner *et al.* [77] have investigated the catalytic performance of compounds of chemical formulae similar to the Schrock catalyst, H-M((NHCH_2_CH_2_)_3_-X) with M = Mo, Ru and Os and X =N and P. The aim of this study was to investigate the catalytic potential of these catalysts for the reduction of N_2_ to NH_3_ using only H_2_ as reducing agent. These calculations were performed with a simplified ligand in which the HIPT substituents are replaced by phenyl groups and the entire ligand is considered at QM/MM level only to estimate its effect on selected properties. DFT calculations revealed that the three compounds possess catalytic cycles for direct N_2_ hydrogenation (Scheme 6), although they show remarkable differences among each other. Indeed, the Mo catalyst is not appropriate due to the high activation energy barriers found for several steps of the catalytic cycle (ΔH^#^_max_ = 69 kcal/mol). The activation free energy barriers found for the other catalysts, bearing Ru and the Os (for the latter the coordinating axial N is replaced by P), are lower (ΔH^#^_max_/ΔG^#^_max_ = 33/43 and 26/36 kcal/mol, respectively). The Os compound showed the smallest ΔG^#^_max_, but this was significantly higher than 30 kcal/mol and thus it was not possible only from theoretical point of view to determine if this catalyst would work experimentally. Interestingly, from this study it appears clearly that the free energy barriers can be tuned by varying transition metals and ligands. 

## 5. Summary and Conclusions

In this review we have presented an overview of computational studies performed to investigate the reaction mechanisms of inorganic complexes able to convert dinitrogen to ammonia. In this respect, particular emphasis is given to the Schrock catalyst, as this is the only inorganic compound known to date catalyzing this process in the presence of a proton and an electron source. A large amount of theoretical studies has been done on this molecule in the last years assessing in detail every reaction step [42,60,63], the importance of the bulky substituents, the influence of solvent for the reaction free energies [42] as well as the importance of level of theory chosen to carry out the study [42,55,57,58]. In addition, Schrock’s mechanism was used as a model to study the reaction mechanisms of other inorganic complexes known to produce ammonia in small yields or simply known to activate N_2_ [18,65,68]. A comparison of their reaction free energies with those of the Schrock catalyst assesses their feasibility to work as potential catalysts for nitrogen fixation. Finally, the theoretical design of clusters mimicking the FeMo-co of nitrogenase is presented [1].

A large amount of literature data is also available for the study of direct N_2_ hydrogenation [72,73,74,75,76] In this respect, dinuclear Chirick-type Zr complex coordinates N_2_ in a side-on mode and converts it to ammonia simply using H_2_ as reducing agent [72,74]. Other studies have been performed with compounds of similar chemical architecture of Schrock’s catalyst, but containing also different transition metals (Ru, Os). For these catalysts the direct conversion of N_2_ to NH_3_ was studied simply using H_2_ as reducing agent [77]. 

Nowadays, DFT can explore quite reliably and relatively quickly the structures and reactivities of transition metal complexes. Therefore, it can be used (i) to explore in detail the reaction mechanisms of known catalysts, (ii) to provide a picture at the molecular level of reaction intermediates, which are not accessible experimentally, (iii) to identify the most energetically demanding steps, (iv) to suggest modifications of the ligands or the use of different transition metals to improve the efficiency of the catalysts; (v) to tailor biomimetic catalysts on the basis of the knowledge of the reaction mechanism of the nitrogenase enzyme.

In conclusion, although DFT calculations provide only an approximate picture of the real systems and they may encounter difficulties in reproducing some energetic or spectroscopic properties, a careful theoretical investigation, which accounts for available experimental data and try to reproduce them as close as possible, can be of crucial help in interpreting experiments, and in complementing them when experimental information is not accessible. Most importantly, all these studies can provide important information to be used by the experimentalists in the development of new efficient catalysts for nitrogen fixation.
